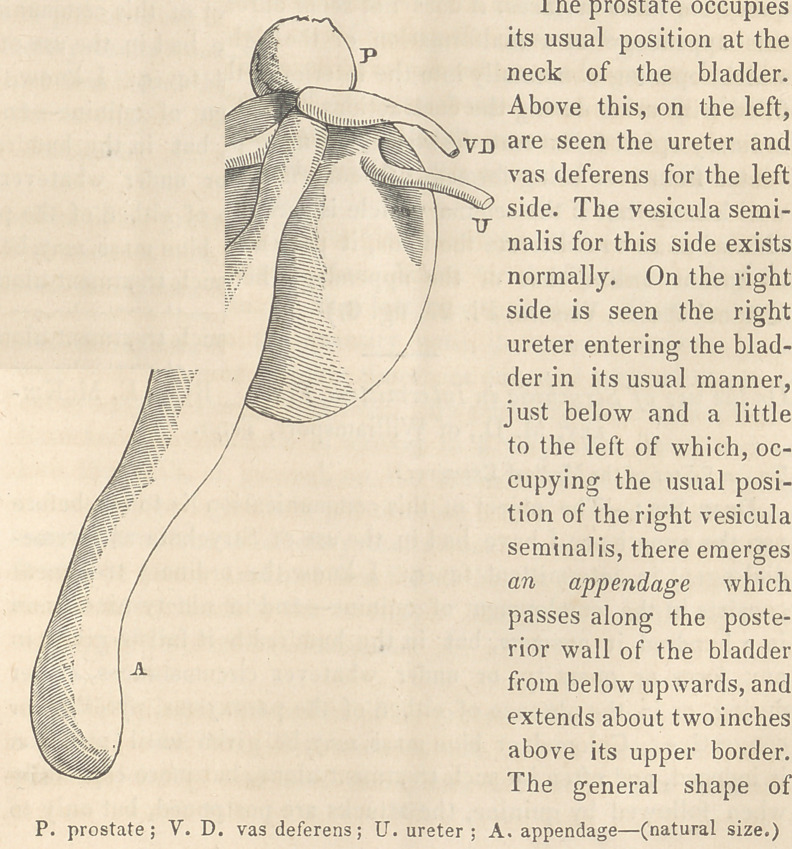# Pathological Cases

**Published:** 1850-07

**Authors:** Carter P. Johnson

**Affiliations:** Professor of Anatomy and Physiology in the Medical Department of Hampden Sidney College, Richmond, Virginia


### MEDICAL EXAMINER,

AND

### RECORD OF MEDICAL SCIENCE.

NEW SERIES.—NO. LXVII.—JULY, 1850.

---

### ORIGINAL COMMUNICATIONS.

---

*Pathological Cases.* By CARTER P. JOHNSON, M. D., Professor of Anatomy and Physiology, in the Medical Department of Hampden Sidney College, Richmond, Virginia.

CASE 1.—*Extensive necrosis and reproduction of the bones of the Fore-arm and Hand.*—The specimen which is the subject of the following description, was presented by Dr. James A. Forbes, of Nelson, Virginia, from whom I obtained these facts. The patient was a negro man, about forty years of age, subject, for a number of years, to epileptiform convulsions. While under the effects of a convulsion, he fell into the fire and burned his right hand and arm and the right side of his face very severely. A high degree of inflammation in the arm and hand followed the accident, and was suffered to run on for the space of twelve months, during which time extensive sloughing of the parts took place, accompanied from time to time by discharge of bone. About a year after the accident, he was seen by Dr. Forbes, and found very much emaciated, and laboring under hectic fever. The arm was immediately amputated a short distance below the elbow, and in fifteen days the patient was walking about. His general health improved very much after the operation, and, singular to say, four years subsequently (the date of the Dr.'s letter to me) he had had *no return of the convulsive disease.*

A ligamentous preparation of the fore arm and hand presents the following appearances. The ulna, throughout its entire extent, is healthy, and does not seem to have participated in the disease. The radius is healthy to within two inches of its inferior extremity, where it becomes enlarged by the deposition of new bone. This new bone, characterized by a peculiar soft and spongy texture, is deposited at its superior limit upon the dorsal portion of the radius, upon and intimately united with the old bone; the amount of new bone increases as you pass down the radius, in the inferior half inch of which it entirely supplants the old bone, and forms the whole of the articulating portion of the carpal extremity of the radius.

In the carpus *no trace of the old bone is discoverable*, each of its eight bones being replaced by a corresponding perfectly formed new bone. In the metacarpus, on the palmar aspect, five newly formed metacarpal bones are seen, all perfect except the anterior portion of that of the thumb; on the dorsal aspect the remaining shells of the old bones of the thumb, index, and middle fingers are seen, being pushed away by the growth of new bone underneath them; the ring finger presents a small sequestrum just making its way through an ulceration in the otherwise perfect involucrum which surrounds it, and in the little finger every particle of the old bone has been replaced by new.

The phalanges of the thumb and index finger, and the second and third phalanges of the middle finger are wanting, (I presume they sloughed away during the progress of the disease. The first phalanges of the middle, the ring, and the little finger are entirely reformed, thin scales of the old bone lying upon their dorsal surfaces, and apparently in the act of being thrown off. The second and third phalanges of the ring finger and the second phalanx of the little finger have been entirely reproduced; the third phalanx of the little finger, which still retains its nail, does not seem to have been materially affected.

In this case we see another illustration of the wonderful reparative power of nature, and of the great results which she is capable of working out, even in a system debilitated by previous disease, when left entirely to her own resources.

Another interesting point connected with the case is the entire cessation of the epileptic attacks subsequent to the accession of the external injury. Did the irritation caused by this extensive disease, which must have been reflected to the nervous centres, excite in the latter a new train of actions preponderating over or breaking up those which had previously given rise to periodical convulsions? And if there is any truth in this vague conjecture, might not the use of *counter irritation* more extensive, and longer continued than usual, merit a trial in these generally unmanageable affections?

Case 2.—*Congenital malformation of the bladder.*—This specimen was presented to me by Dr. R. W. Haxall, of this city, and was obtained from a white male child who died at the age of about eight months, having suffered violent paroxysms of pain in the hypogastric and umbilical regions for several weeks previously.

The bladder is of the natural size and form, and viewed on its anterior aspect presents no unusual appearance. Viewed from behind, the following condition of things, accurately represented in the accompanying diagram, is observed.

The prostate occupies its usual position at the neck of the bladder. Above this, on the left, are seen the ureter and vas deferens for the left side. The vesicula seminalis for this side exists normally. On the right side is seen the right ureter entering the bladder in its usual manner, just below and a little to the left of which, occupying the usual position of the right vesicula seminalis, there emerges *an appendage* which passes along the posterior wall of the bladder from below upwards, and extends about two inches above its upper border. The general shape of

P. prostate; V. D. vas deferens; U. ureter; A. appendage—(natural size.)

this appendage is irregularly cylindrical; attaching itself by a small pedicle of about four lines in diameter to the bladder, it very soon enlarges to a diameter of about ten lines, which it maintains throughout its course.

On examining the interior of the bladder, this appendage, which is hollow throughout, is found to open by a contracted orifice just within and below the orifice of the right ureter. Its structure is the same as that of the bladder, of which it forms a simple diverticulum. When the body was opened it was found distended with urine.

Have the congenital absence of the right vesicula seminalis and the existence of this singular diverticulum any relation to each other, or are they to be regarded as simple coincidences? Knowing, as we now do, that all glands whose ducts open upon either of the great mucous surfaces, are originally developed as diverticula from these surfaces, it does not seem unreasonable to regard this appendage as a malformation of the right seminal vesicle, which, opening abnormally into the interior of the bladder, became filled with urine during the contractions of that organ, whereby its ordinary spiral folds were destroyed, or, more probably, were prevented from ever being formed. In confirmation of this view, it is well known that if the seminal vesicle is carefully dissected out and inflated so as to obliterate the folds, it presents an appearance by no means unlike that of the appendage here described. (See Quains' Plates, Viscera, Pl. 27, fig. 6.)

## Figures and Tables

**Figure f1:**